# Single Source Precursor-based Solvothermal Synthesis of Heteroatom-doped Graphene and Its Energy Storage and Conversion Applications

**DOI:** 10.1038/srep05639

**Published:** 2014-07-10

**Authors:** Bo Quan, Seung-Ho Yu, Dong Young Chung, Aihua Jin, Ji Hyun Park, Yung-Eun Sung, Yuanzhe Piao

**Affiliations:** 1 Graduate School of Convergence Science and Technology, Seoul National University, Seoul 151-742, Republic of Korea; 2 Center for Nanoparticle Research, Institute for Basic Science (IBS), Seoul 151-742, Republic of Korea; 3 School of Chemical and Biological Engineering, Seoul National University, Seoul 151-742, Republic of Korea; 4 Research Institute of Advanced Materials (RIAM), Seoul National University, Seoul 151-742, Republic of Korea; 5 Advanced Institutes of Convergence Technology, Suwon 443-270, Republic of Korea

## Abstract

Solvothermal processes are considered efficient approaches for the gram-scale production of graphene. Further modification of graphene by chemical doping is an important approach to tailor its properties. In this work, we successfully synthesized sulfur-doped graphene by using a solvothermal method with dimethyl sulfoxide as a precursor, which is a common laboratory reagent. Nitrogen-doped graphene was produced to demonstrate the generality of this process. These heteroatom-doped graphene materials exhibited high surface areas and high contents of heteroatoms. Furthermore, the lithium-ion storage properties and oxygen reduction reaction catalytic activity of these materials were also investigated. The success of this approach might facilitate the development of other advanced graphene-based materials with relative simplicity, scalability, and cost effectiveness for use in various potential applications.

Graphene has attracted great interest in many research areas because of its unique structure and outstanding properties^1^, e.g., two-dimensional structured graphene has been widely studied for its use in electronics^2^, nanomedicine^3^, sensors^4^, catalysts^5^, supercapacitors^6^, and lithium ion batteries^7^,^8^. To explore novel functions and potential applications of graphene, the morphology was tailored to obtain various types of graphene architectures, such as zero-dimensional graphene quantum dots (GQDs)^9^,^10^, two-dimensional graphene nanoribbons (GNRs)^11^, and three-dimensional graphene structures^12^,^13^,^14^. Chemical doping is considered another effective approach to tailoring the electrical properties and chemical activities of graphene since its spin density and atomic charge density will be influenced by the dopants^15^.

Various elements (B, N, P, S, etc.) were selected in order to introduce into carbon frameworks to produce heteroatom-doped graphene^15^,^16^,^17^,^18^,^19^. In previous studies, N-doped graphene has been produced by various approaches that can be categorized into two kinds of methods: direct synthesis such as chemical vapor deposition (CVD), arc-discharge, segregation growth, and solvothermal methods; and post treatment such as thermal, hydrazine hydrate, and plasma treatments^15^,^20^,^21^,^22^. N-doped graphene exhibits different properties according to their synthetic approaches because their N-doping content and N-doping types are different. For the efficient preparation of S-doped graphene, graphene oxide can be thermally treated with a sulfur source. Various sulfur containing molecules (SO_2_, H_2_S, CS_2_, and benzyl disulfide) were studied as sources, and the catalyst activities of S-doped graphene for the oxygen reduction reaction (ORR) were investigated^17^,^23^,^24^,^25^. However, developing a simple, low-cost, large-scale production of heteroatom-doped graphenes remains an important challenge. Additionally, each element requires a different synthetic doping method^15^,^24^. Stride and co-workers demonstrated that graphene can be prepared by using a solvothermal method with ethanol as the carbon source^26^, and the reaction is relatively mild and simple^27^. Furthermore, it provides for a gram-scale production of graphene.

In this research, we describe a novel approach to fabricate S-doped graphene via a solvothermal method using S-containing organic molecules as a precursor. This method directly converts dimethyl sulfoxide (CH_3_CH_3_SO, DMSO) into S-doped graphene. Furthermore, N-doped graphene was also synthesized using dimethylformamide (CH_3_CH_3_NCOH, DMF), which is a N-containing organic molecule. These precursors are commonly used as low-cost solvents. It provides the benefits of a single step reaction with relatively mild synthesis conditions, gram-scale products, and higher contents of the heteroatom. The lithium-ion storage properties and electrocatalytic behaviors of heteroatomic-doped graphene were investigated.

## Results

### Synthesis and characterization of heteroatom-doped graphene

**Figure 1:**
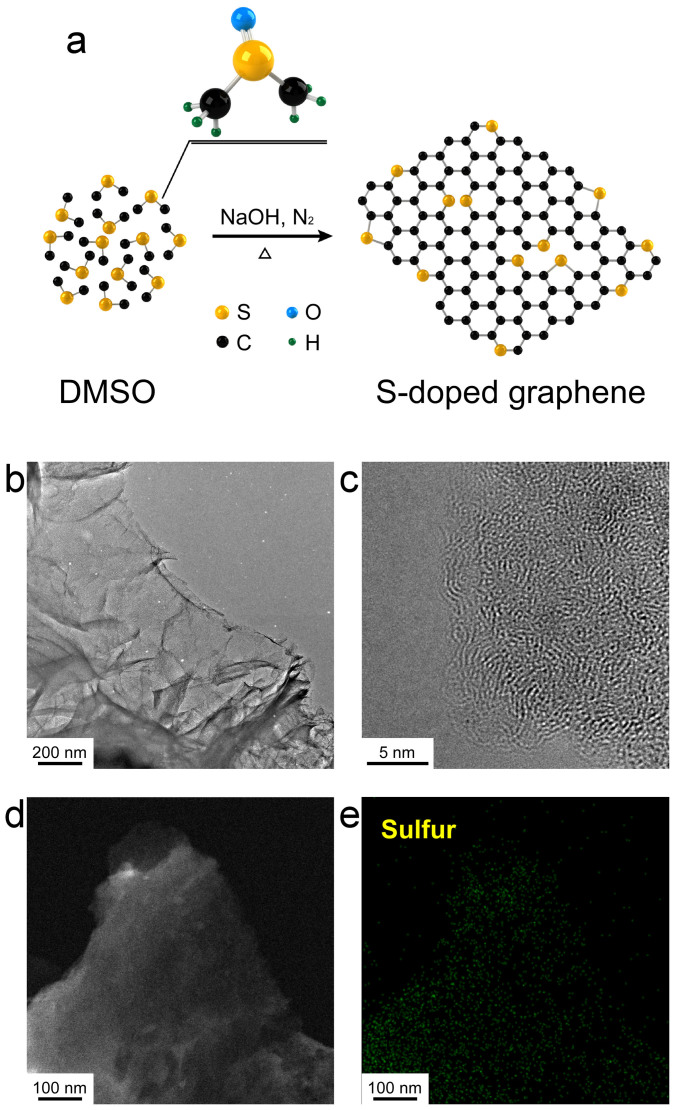
Synthesis of the S-doped graphene. (a) Schematic illustration of the formation of the S-doped graphene. (b) TEM image of the S-doped graphene, (c) high-resolution TEM image of the S-doped graphene, (d) dark-field TEM image of the S-doped graphene, and (e) sulfur elemental mapping of the S-doped graphene.

DMSO, the S-containing organic molecule, was heated with NaOH under N_2_ gas flow. The mixture was brought to a boil, and maintained the boil with reflux. Under the condition, the colorless liquid became dark brown (see Supplementary Fig. S1). Finally, black cake-like materials were obtained and washed using deionized water then dried in an oven. Two-dimensional sheet-like structures were obtained, which were formed with carbon and sulfur atoms (Fig. 1). The surface morphology of the S-doped graphene was analyzed by using atomic force microscopy (AFM). The AFM images showed crumpled silk veil-like structures with thickness of around 1 nm (see Supplementary Fig. S2). With 50 ml of DMSO, more than 1.0 g of product per batch was obtained. We believe that this process could be scaled up for larger synthetic yields. To demonstrate that the heteroatom-containing organic molecules could be converted into heteroatom-doped graphene, DMF was chosen to produce N-doped graphene, to represent N-containing molecules. Roughly 2.6 g of product was obtained from 50 mL of DMF. Pristine solvothermal graphene was also prepared using methanol as a precursor (see Supplementary Fig. S3). In a previous study, hexagonal carbon clusters were synthesized by using alkali metals as reducing agents at a low temperature^28^. In that study, free C = C was stated to be the intermediate during the formation of sp^2^ hybridized carbon. N-doped graphene was also synthesized through the reaction of alkali metal salts (Li_3_N) and carbon source (CCl_4_)^29^. Therefore, we propose that C = C, C-S-C, and C-S groups are probably the reaction intermediates, which subsequently assemble into a S-contained sp^2^ hybridized carbon (Fig. 1a).

**Figure 2:**
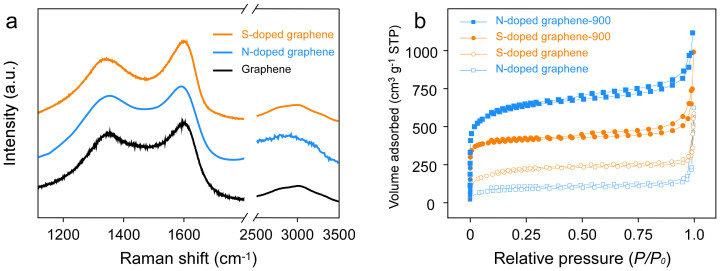
Raman and surface area characterization. (a) Raman spectrum of the S-doped graphene, N-doped graphene, and solvothermal graphene. (b) N_2_-adsorption/desorption of the S-doped graphene, N-doped graphene, S-doped graphene-900 (annealing at 900°C), and N-doped graphene-900 (annealing at 900°C).

To investigate the elemental dopant distribution, TEM energy-dispersive X-ray spectroscopy (EDS) mapping was carried out. Fig. 1d shows a homogeneous distribution of elemental S in the graphene sheets. Raman spectroscopy of S-doped graphene, N-doped graphene, and solvothermal graphene was investigated. Broad D and G bands were determined at around 1350 and 1600 cm^−1^, respectively (Fig. 2). The spectra of as-synthesized graphene agreed well with the literature^26^. X-ray diffraction (XRD) patterns of the solvothermal graphene and graphite were analyzed (Fig. S4). Graphite shows a sharp peak at 26.3° (see Supplementary Fig. S4) corresponding to the (002) diffraction peak (d-spacing = 0.34 nm). The (002) peaks of the S-doped graphene, N-doped graphene and solvothermal graphene are shown at 22.3°, 25.5°, and 23.4°, respectively. The interlayer distance of the S-doped graphene is 0.40 nm, which is larger than that of the N-doped graphene (0.35 nm) and solvothermal graphene (0.38 nm). The conductivity of the S-doped graphene and N-doped graphene is 0.607 × 10^−3^ S m^−1^ and 0.416 × 10^−3^ S m^−1^, which is lower than that of the solvothermal graphene (0.121 S m^−1^; see Supplementary information). The results show that S or N doping increases the resistance of graphene.

The N_2_ adsorption and desorption isotherms and pore size distributions of the heteroatom-doped graphene were also analyzed (Fig. 2b and Supplementary Fig. S5). The Brunauer–Emmett–Teller (BET) surface area of the S-doped graphene and N-doped graphene were 754 m^2^ g^−1^ and 317 m^2^ g^−1^ with the pore volumes (PVs) of 0.82 cm^3^ g^−1^ and 0.91 cm^3^ g^−1^, respectively. After annealing at high temperature (900°C) under an inert atmosphere, the respective BET surface areas of the S-doped graphene and N-doped graphene were increased to 1564 m^2^ g^−1^ (PV = 1.53 cm^3^ g^−1^) and 2255 m^2^ g^−1^ (PV = 1.73 cm^3^ g^−1^).

**Figure 3:**
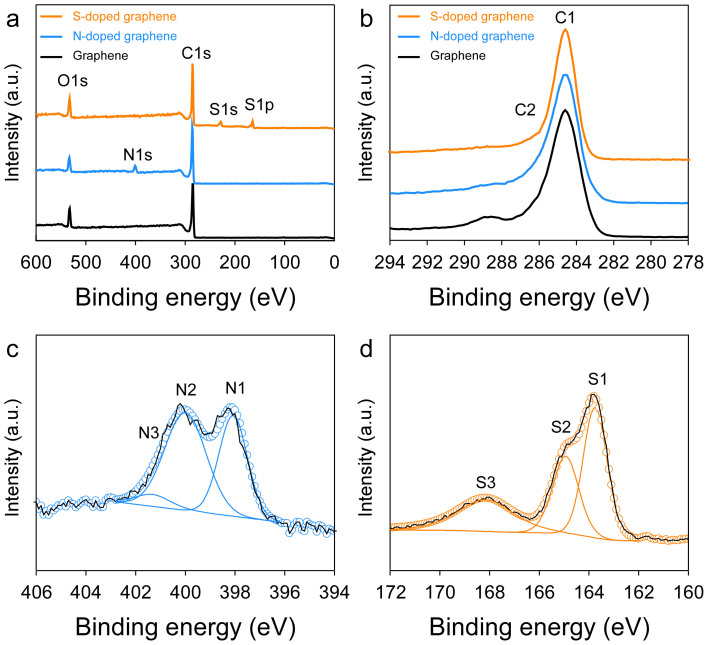
XPS spectra. (a) XPS spectra of the S-doped graphene, N-doped graphene, and solvothermal graphene. (b) C1s XPS spectra of the S-doped graphene, N-doped graphene and solvothermal graphene. (c) High resolution S2p XPS spectra of the S-doped graphene with S1 (S2p_3/2_), S2 (S2p_1/2_), and S3 (oxidized sulfur). (d) High resolution N1s XPS spectra of the N-doped graphene with N1 (pyridinic-N), N2 (pyrrolic-N), and N3 (graphitic-N).

For elucidating the chemical structure of the S-doped graphene, X-ray photoelectron spectroscopy (XPS) was employed. The XPS spectra of the solvothermal graphene derived from methanol confirmed that carbon and oxygen were the only atoms present. However, sulfur and nitrogen signals could be clearly observed in the XPS spectra of the S-doped graphene and N-doped graphene, respectively (Fig. 3a). The high-resolution C1s peak corresponding to sp^2^-hybridization (C-C bond, 284.5 eV) was observed, and the samples produced a small peak corresponding to C-O bonds, carbonyls (C = O), and carboxylates (O-C = O) (Fig. 3b)^30^. The high-resolution S2p spectra of the S-doped graphene can be deconvoluted into three different peaks (Fig. 3c). Lines at 163.9, 165.1 and 168.5 eV correspond to 2p_3/2_, 2p_1/2_, and oxidized sulfur groups, respectively. Additionally, there existed an invisible peak corresponding to the thiol group (162.0 eV)^24^. The high-resolution N1s spectra of the N-doped graphene can also be decomposed into three different peaks that can be ssigned to pyridinic N (398.0 eV), pyrrolic N (400.1 eV), and graphitic N (401.3 eV) (Fig. 3d)^24^. The results suggest that the S or N atoms are covalently bonded network of graphene. According to the elemental analysis, the sulfur content of the S-doped graphene was 22.83 wt%, and the nitrogen content of the N-doped graphene was 12.25 wt% (see Supplementary Table S1). The sulfur content of the S-doped graphene is much higher than those previously reported literature (<5%)^17^,^24^.

### Lithium storage properties

**Figure 4:**
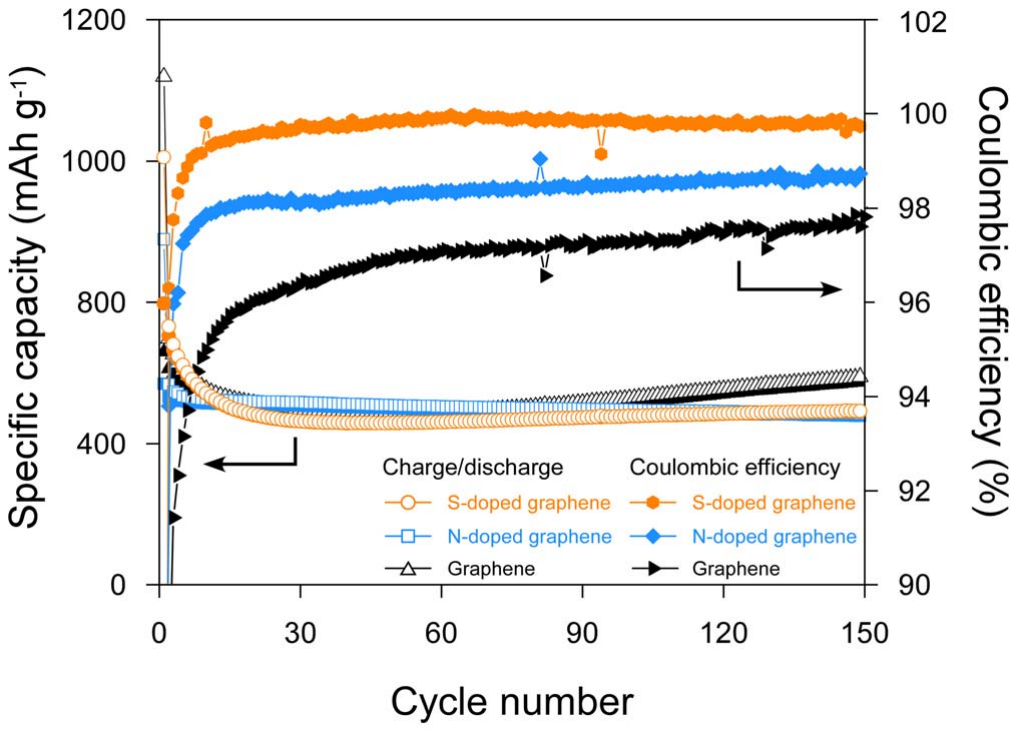
Lithium storage properties. Cycle performances and Coulombic efficiencies of the S-doped graphene, N-doped graphene, and solvothermal graphene at a current density of 200 mA g^−1^.

To the best of our knowledge, the battery properties of the graphene family prepared by solvothermal methods have yet to be reported. To evaluate the potential applicability of the S-doped graphene, N-doped graphene and solvothermal graphene as anode materials, electrochemical cells with graphene electrodes were galvanostatically charged and discharged at a current density of 200 mA g^−1^ at room temperature (Fig. 4). The reversible capacities of the S-doped graphene, N-doped graphene and solvothermal graphene electrodes are reasonably high (500–800 mAh g^−1^). Interestingly, the specific capacities decrease during initial 30 cycles and gradually increase as the number of cycles increases in the S-doped graphene and solvothermal graphene. These activation processes can also be observed in other graphene-based electrodes^31^,^32^. Notably, the S-doped graphene, N-doped graphene and solvothermal graphene electrodes exhibited high cyclic stability without the capacity fluctuations that might occur during long cycles, which has also been observed in other reported graphene electrodes^11^,^33^. The initial Coulombic efficiencies of the S-doped graphene, N-doped graphene and solvothermal graphene are 65.8%, 58.3%, and 45.8%, respectively. It is worthwhile to comment that the average Coulombic efficiencies of solvothermal graphene from the 30_th_ to 150_th_ cycle was 97.2%, which is a relatively high value, but those of the S-doped and N-doped graphene were much higher (99.8% and 98.5%, respectively). It was demonstrated that half-cell Coulombic efficiency could affect cycle retention in full lithium ion cells^34^. Additionally, the plateau in the first discharge process that occurs around 1.0–0.7 V in voltage profiles (more clearly seen in the CV curves) is related to the decomposition of electrolyte and the formation of a solid electrolyte interphase (SEI) layer (see Supplementary Fig. S6)^31^,^32^, this plateau is remarkable in the solvothermal graphene electrode. These findings indicate that the doping of nitrogen and sulfur in graphene can suppress side reactions, which include electrolyte decomposition^11^. The shape of charge and discharge profiles in the N-doped graphene and solvothermal graphene is similar to those in other graphene^11^,^32^; however, the S-doped graphene profile is slightly different. Additional cathodic (~1.4 V) and anodic (~2.4 V) peaks were observed in the CV curves of the S-doped graphene (see Supplementary Fig. S6e), which are attributed to the conversion of polysulfides to lithium sulfides and lithium sulfides to polysulfides, respectively^31^. The N-doped graphene electrode shows the highest rate capability. The S-doped graphene, N-doped graphene, and solvothermal graphene electrodes delivered specific capacities of 270.5, 278.9, and 267.5 mAh g^−1^, respectively, at current density of 2500 mA g^−1^, which correspond to the respective delivered capacities of 44.9%, 40.7%, and 53.1% at a current density of 200 mA g^−1^. The theoretical capacity of graphene is 744 mAh g^−1^ through the formation of LiC_3_ in a condition where all the graphene layers present as monolayers^35^,^36^. It is reported that the lithium storage properties of graphene have a sensitive dependence on many factors such as synthetic methods and condition, interlayer spacing, disorder degree, and surface functional groups. The reversible capacities of graphene can vary from around 300 mAh g^−1^ to more than 1500 mAh g^−1^ (see Supplementary Table S2)^19^,^32^,^37^,^38^,^39^. The S-doped graphene and N-doped graphene by solvothermal method display high cycle stability with excellent Coulombic efficiency compared to other previous reported graphene electrodes.

### Electrocatalytic performance

**Figure 5:**
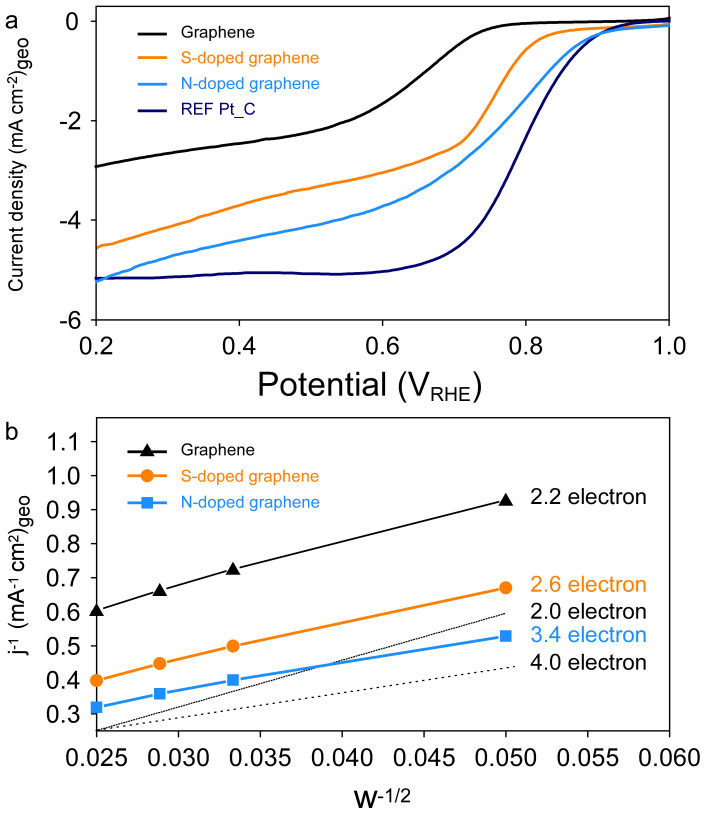
Electrocatalytic behaviors. (a) RDE polarization curves in O_2_ saturated 0.1 M KOH (rotation speed, 1600 rpm; scan rate, 5 mV s^−1^) and (b) K-L plots of the samples derived from RDE data at different rotating speeds (0.6 V_RHE_).

To investigate the electrocatalytic activity of the S-doped graphene and N-doped graphene for the ORR and to confirm the doping effect, we performed rotating-disk electrode (RDE) voltammetry in 0.1 M KOH electrolyte (20 wt% Pt/C commercial E-TEK catalyst). The N-doped graphene shows higher ORR activity than pristine solvothermal graphene. Further, N-doped graphene has a similar onset potential (0.9 V_RHE_) as platinum electrode. The activity of doped carbon for the ORR has been improved by a combination of many factors, which have not yet been clarified^40^,^41^,^42^. However, it is important to note that the enhancement factor can be explained by doping, for example, the presence of N, B, and S in carbon^17^,^43^,^44^,^45^. These results indicate that the electrocatalytic activities of the catalyst for the ORR increase with the introduction of a dopant whose electronegativity is different from that of carbon. Furthermore, Koutechy-Levich analysis by changing the rotating speed reveal that the electron transfer number changes from 2.8 in the pristine solvothermal graphene to 3.9 in the N-doped graphene at 0.2 V_RHE_ (see Supplementary Fig. S7). For clear emphasis on the importance of proper doping effects on ORR activity in the region of real application, we chose the potential (0.6 V_RHE_) to compare the electron transfer number in Fig. 5b, which is very high potential compared to the general viewpoint^44^. In the case of solvothermal graphene, the electron transfer number indicates dominant two-electron pathway that is ineffective for the ORR. However, when doping with elements such as N and S, the ORR pathway would involve both 2-electron and 4-electron transfer which are more favorable for the ORR. Solvothermal heteroatom (N, S)-doped graphene was synthesized by a bottom-up method. It is different from top-down method and contains a significant amount of metallic impurities which may influence the activity^46^,^47^. In this work, our solvothermal process uses NaOH or Na metals which do not affect the activity. XPS results also confirmed that there is no other impurity in the as-synthesized graphene. Considering that the doping content in the present metal-free graphene is rather optimized, further improvement of ORR activity can be achieved by optimizing the contents and configuration.

## Discussion

In summary, heteroatom (S or N)-doped graphenes were successfully synthesized by using a simple, single-step solvothermal method with heteroatom-containing organic molecules as precursors. This process provides for a gram-scale production using common laboratory reagents. The solvothermal (S or N)-doped graphenes exhibited high heteroatom content and surface area, and their characterization data indicate that they could be utilized in various energy storage and conversion applications. We believe that heteroatom-doped graphenes can be further extended to applications in sensors, electronics and biological research. We expect that our synthetic approach will be developed to produce doped carbon materials based on other elements (e.g., B, P, and F) which can then increase the method's potential applications.

## Methods

### Synthesis of S-doped graphene

A mixture of 2 g of NaOH and 10 mL of DMSO solution was added into a three-neck flask with a reflux condenser and heated under a N_2_ gas flow. The colorless mixture was boiled at around 250°C and was slowly thickened. After boiling for ~24 h, the liquid was converted into a black cake-like substance. Finally, the samples were washed with hydrochloric acid (10 wt%) and deionized water several times, and then dried in an oven at 90°C. The final yield of S-doped graphene is ~35 mg per 1 ml of DMSO (see Supplementary Fig. S1).

### Synthesis of N-doped graphene

N-doped graphene was synthesized by using a solvothermal method^26^. In a typical synthesis, 50 mL of DMF and 25 g of sodium metal were placed in a 100 mL Teflon lined autoclave that was sealed and heated at 190°C for 72 h. After cooling to room temperature, the autoclave was opened. The product was removed immediately to a beaker containing 10 wt% of hydrochloric acid. The sample was further washed with deionized water several times, and dried in an oven at 90°C. (Caution: before washing, the sodium-containing solid state sample can catch fire easily when exposed to air.)

### Synthesis of solvothermal graphene

Solvothermal graphene were prepared according to a literature procedure using methanol as the precursor^26^. In a typical synthesis, 50 mL of methanol and 25 g of sodium metal were placed in a 100 mL Teflon lined autoclave that was sealed and heated at 190°C for 72 h. After the autoclave was allowed to cool to room temperature, the solids were annealed at 500°C under a N_2_ gas flow. The resulting samples were washed with hydrochloric acid (10 wt%) and deionized water several times, and then dried in an oven at 90°C.

### Characterization

TEM images and TEM-EDS images were obtained using a JEOL JEM-1010 and JEM-2100F, respectively. AFM images were obtained using a Veeco atomic force microscope. Raman spectroscopy measurements were carried out on a Dongwoo DM500i Raman spectrometer using green (514.5 nm) laser excitation. BET surface area and BJH pore size distributions were measured by using a Micromeritics Tristar 3000. XPS was carried out using a Kratos AXIS-HSi. Elemental analysis was performed using a Thermo Scientific Flash 2000 analyzer at NCIRF (National Center for Inter-university Research Facilities, Seoul National University, Korea).

### Electrochemical characterization (battery application)

Samples were prepared by heat treatment at 600°C in N_2_ for battery applications. For the preparation of working electrodes, a well-mixed slurry of synthesized graphene, Super P and poly (vinylidene fluoride) in N-methyl-2-pyrrolidone (at a weight ratio of 70:10:20) was coated on a copper current collector. After drying, the electrodes were pressed and dried again at 120°C under vacuum. Two electrode 2016-type coin cells were fabricated with prepared working electrodes and lithium foil (as both counter and reference electrodes) in argon filled glove box. 1.0 M LiPF_6_ dissolved in ethylene carbonate (EC) and diethyl carbonate (DEC) (1:1 in volume ratio) was used as the organic electrolyte. All the voltage ranges for the battery tests in this study were set between 3.0 and 0.01 V (vs. Li^+^/Li). The specific capacities were calculated only according to the weight of the active material and the weights of additives (binder and conductive agent) were not included.

### Electrochemical characterization (electrocatalytic behavior)

Samples were prepared by heat treatment at 600°C in N_2_ for electrocatalytic applications. Electrochemical measurements were conducted using an Autolab PGSTAT 101 in a standard three-compartment electrochemical cell. In all experiments, a Ag/AgCl reference electrode was used as the reference electrode, and Pt wire was used as the counter electrode. All potentials referred to in these ORR experiments were converted to the pH-independent reversible hydrogen electrode (RHE) using the hydrogen oxidation reaction. Three kinds of graphene samples (5 mg) and 20 μL of 5 wt% Nafion ionomer were each dispersed 0.4 mL of 2-isopropyl alcohol by sonication for 10 min. For RDE, 7 μL of catalyst ink was loaded onto an RDE with a 0.000196 m^2^ glassy carbon disk. The electrode rotation speed was 1600 rpm (scan rate, 5 mV s^−1^), 0.1 M KOH electrolyte. To calculate the electron transfer number (0.6 V_RHE_), RDE measurements were conducted using different rotating speeds 400, 900, 1200, and 1600 rpm.

## Author Contributions

B.Q. proposed the concept and performed the experiment. S.-H.Y. and A.J. carried out lithium storage properties. D.Y.C. carried out electrocatalytic performances. J.H.P. carried out the AFM data. Y.P., B.Q. and S.-H.Y. co-wrote the manuscript. Y.P. and Y.-E.S. coordinated and supervised the work.

## Supplementary Material

Supplementary Information Single Source Precursor-based Solvothermal Synthesis of Heteroatom-doped Graphene and Its Energy Storage and Conversion Applications
