# Supplementary material for: Single Source Precursor-based Solvothermal Synthesis of Heteroatom-doped Graphene and Its Energy Storage and Conversion Applications
Source: Sci Rep. 2014 Jul 10;4:5639. doi: 10.1038/srep05639 (PMC4090616; doi:10.1038/srep05639)
Supplement: Supplementary Information — Single Source Precursor-based Solvothermal Synthesis of Heteroatom-doped Graphene and Its Energy Storage and Conversion Applications [file srep05639-s1.pdf]

Supplementary information

Single Source Precursor-based Solvothermal Synthesis of Heteroatom-doped Graphene and Its Energy Storage and Conversion Applications

Bo Quan[1], Seung-Ho Yu[2,3,4], Dong Young Chung[2,3], Aihua Jin[2,3], Ji Hyun Park[1], Yung-Eun Sung[2,3*] & Yuanzhe Piao[1,5*]

[1]Graduate School of Convergence Science and Technology, Seoul National University, Seoul 151-742, Republic of Korea.

[2]Center for Nanoparticle Research, Institute for Basic Science (IBS), Seoul 151-742, Republic of Korea.

[3]School of Chemical and Biological Engineering, Seoul National University, Seoul 151-742, Republic of Korea.

[4]Research Institute of Advanced Materials (RIAM), Seoul National University, Seoul 151-742, Republic of Korea.

[5]Advanced Institutes of Convergence Technology, Suwon 443-270, Republic of Korea.

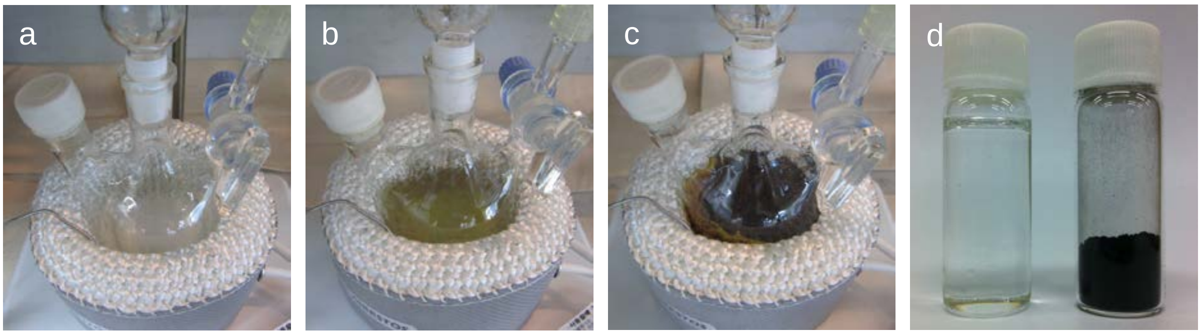

Supplementary Figure S1: Photographs of synthesis of the S-doped graphene. (a) A mixture of DMSO and NaOH in a reactor (room temperature). (b) The mixture started to boil at ~250 °C, and (c) the mixture was boiling about 1 h. (d) 10 ml of DMSO and 0.35 g of the S-doped graphene.

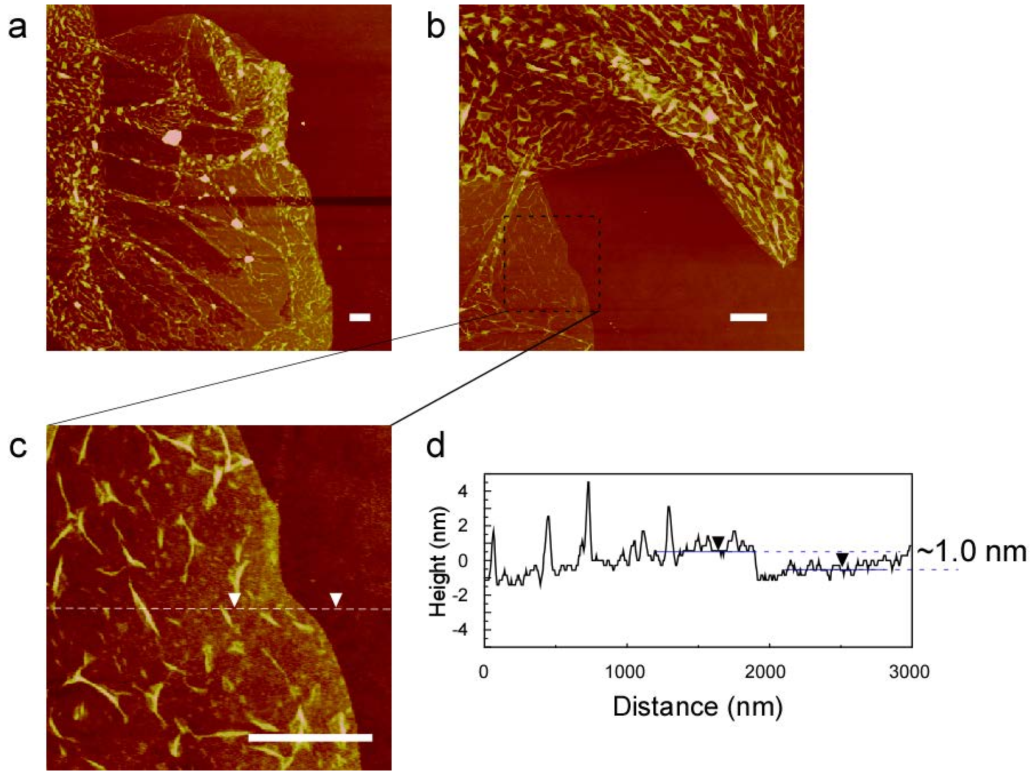

Supplementary Figure S2: AFM images of the S-doped graphene. (a, b, and c) AFM images of the S-doped graphene with different magnifications (All the scale bars are 1000 nm.) (d) Height profile of the S-doped graphene.

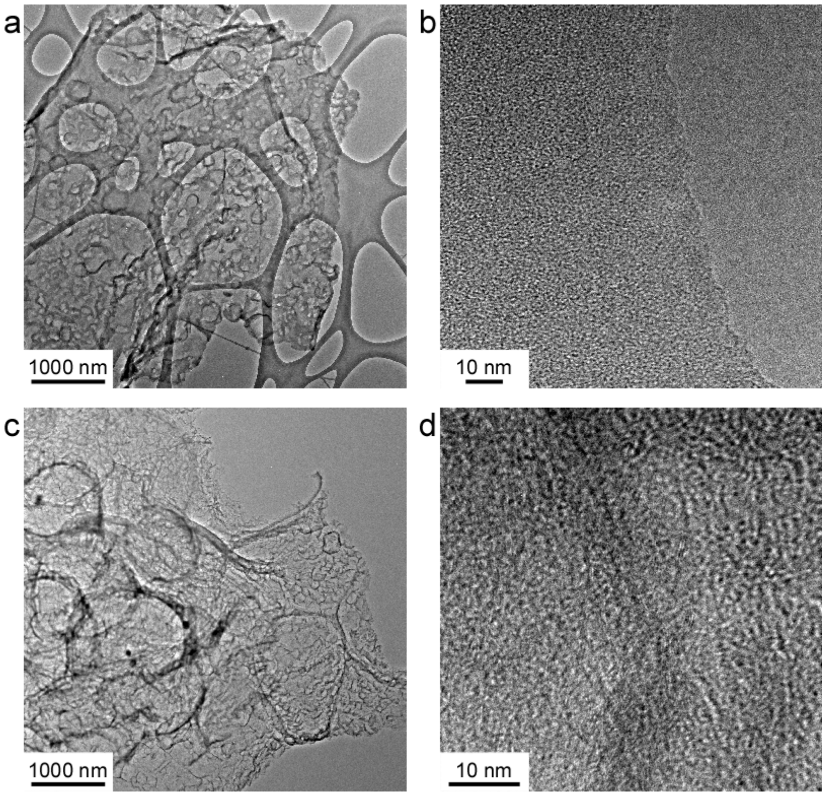

Supplementary Figure S3: TEM and high-resolution TEM images of the N-doped graphene and pristine solvothermal graphene. (a, b) TEM and HR- TEM images of the N-doped graphene. (c, d) TEM and HR-TEM images of the pristine solvothermal graphene.

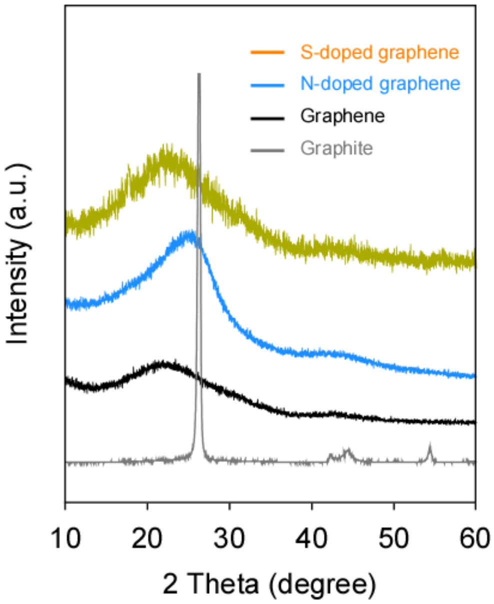

Supplementary Figure S4: XRD patterns of the S-doped graphene, N-doped graphene, pristine solvothermal graphene, and graphite.

Bulk conductivity

Bulk conductivity of the S-doped graphene, N-doped graphene and pristine solvothermal graphene were measured according to a literature procedure[1]. The samples were filled into a channel; two sides of the channel are used as electrodes. The conductivity of samples was measured using the equation: $\sigma = R \cdot L / A$

Where:

σ is the conductivity, Sm^{-1}

R is the resistance, Ω

L is the distance between the two electrodes, m

A is the cross-section area of the sample, m^2

**Sample**	**Conductivity (S m^{-1})**
**S-doped graphene**	0.607×10^{-3}
**N-doped graphene**	0.416×10^{-3}
**Pristine graphene**	0.121
**Pristine graphene[20]**	0.067

20 Choucair, M., Thordarson, P. & Stride, J. A. Gram-scale production of graphene based on solvothermal synthesis and sonication. *Nat. Nanotechnol.* **4**, 30-33 (2009).

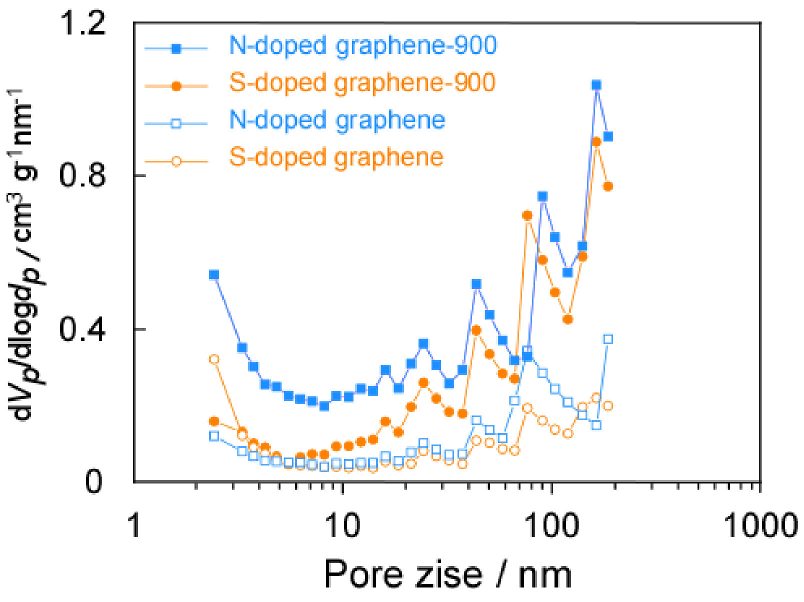

Supplementary Figure S5: Pore size analysis. Pore distributions of the S-doped graphene, N-doped graphene, S-doped graphene-900 (annealing at 900 °C), and N-doped graphene-900 (annealing at 900 °C).

Supplementary Table S1: Elemental analysis data of the S-doped graphene, N-doped graphene and pristine solvothermal graphene.

	% Composition/Mass				
**Sample**	**C**	**H**	**N**	**S**	**O**
**S-doped graphene**	70.13	1.49	0.08	22.83	5.47
**N-doped graphene**	65.41	2.40	12.25	-	19.94
**Pristine graphene**	88.41	0.99	0.09	-	10.51
**Pristine graphene[20]**	78.49	2.69	-	-	18.82

A previous study demonstrated that most of the oxygen present in solvothermal graphene was adsorbed water[20].

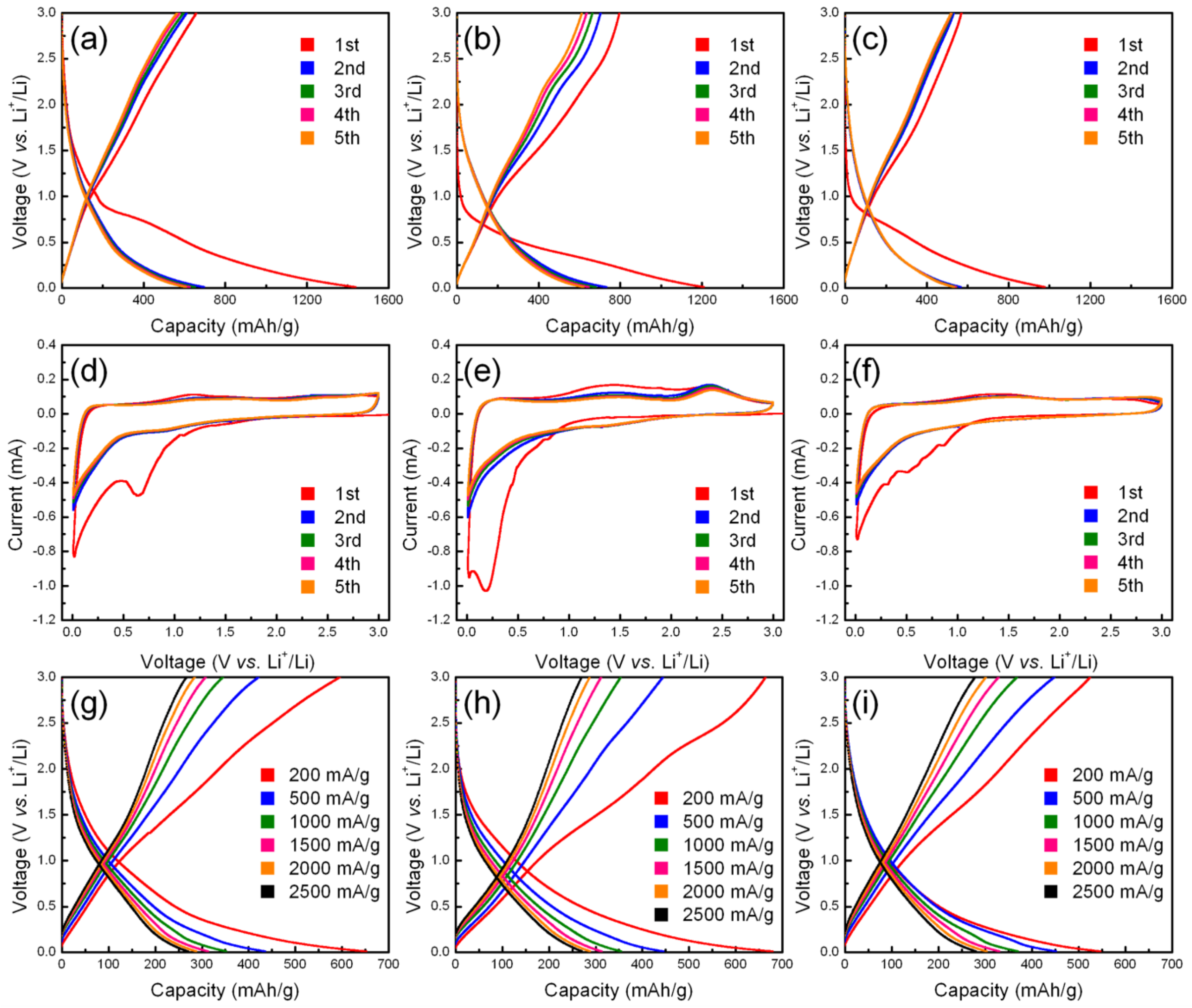

Supplementary Figure S6: Galvanostatic charge and discharge profiles of (a) the pristine solvothermal graphene, (b) S-doped graphene and (c) N-doped graphene electrodes at a current density of 200 mA g^{-1} for initial 5 cycles. Cyclic voltammograms of (d) the pristine solvothermal graphene, (e) S-doped graphene and (f) N-doped graphene electrodes at a scanning rate of 0.1 mV s^{-1}. Galvanostatic charge and discharge profiles of (g) the pristine solvothermal graphene, (h) S-doped graphene and (i) N-doped graphene electrodes at various current rates.

Supplementary Table S2: Reversible capacities of graphene as anode materials.

sample	Current density	Voltage range (*vs.* Li^+/Li)	Initial reversible capacity	Reversible capacity after cycles	Ref.
GO annealed at 600 ºC	50 mAh g^{-1}	0.005 – 3.0 V	794 mAh g^{-1}	~700 mAh g^{-1} (15th cycle)	30
GO annealed at 300 ºC	50 mAh g^{-1}	0.005 – 3.0 V	1013 mAh g^{-1}	834 mAh g^{-1} (15th cycle)	30
Electron beam reduced GO	50 mAh g^{-1}	0.005 – 3.0 V	1054 mAh g^{-1}	784 mAh g^{-1} (15th cycle)	30
Hydrazine reduced GO	50 mAh g^{-1}	0.005 – 3.0 V	330 mAh g^{-1}	~200 mAh g^{-1} (15th cycle)	30
GO annealed at 1050 ºC	100 mAh g^{-1}	0.01 – 3.0 V	~400 mAh g^{-1}	~270 mAh g^{-1} (100th cycle)	26
GO annealed at 1050 ºC + Heat treatment in NH_3 (N-doped graphene)	100 mAh g^{-1}	0.01 – 3.0 V	~500 mAh g^{-1}	~360 mAh g^{-1} (17th cycle), ~450 mAh g^{-1} (100th cycle), ~680 mAh g^{-1} (500th cycle)	26
GO annealed at 1000 ºC with triphenylmethane	100 mAh g^{-1}	0.01 – 3.0 V	280 mAh g^{-1}	~280 mAh g^{-1} (80th cycle)	14
GO annealed at 1000 ºC with triphenylphosphine (P-doped graphene)	100 mAh g^{-1}	0.01 – 3.0 V	460 mAh g^{-1}	~460 mAh g^{-1} (80th cycle)	14
Hydrazine reduced GO	50 mAh g^{-1}	0.01 – 3.5 V	540 mAh g^{-1}	290 mAh g^{-1} (20th cycle)	31
GO annealed at 500 ºC	50 mAh g^{-1}	0.01 – 3.0 V	955 mAh g^{-1}	638 mAh g^{-1} (30th cycle)	32
GO annealed at 600 ºC in NH_3 (N-doped graphene)	50 mAh g^{-1}	0.01 – 3.0 V	1043 mAh g^{-1}	873 mAh g^{-1} (30th cycle)	32
GO annealed at 800 ºC in BCl_3 (B-doped graphene)	50 mAh g^{-1}	0.01 – 3.0 V	1549 mAh g^{-1}	1227 mAh g^{-1} (30th cycle)	32
Solvothermal synthesis using methanol (Pristine graphene)	200 mAh g^{-1}	0.01 – 3.0 V	660 mAh g^{-1}	577 mAh g^{-1} (150th cycle)	this work
Solvothermal synthesis using DMF (N-doped graphene)	200 mAh g^{-1}	0.01 – 3.0 V	797 mAh g^{-1}	479 mAh g^{-1} (150th cycle)	this work
Solvothermal synthesis using DMSO (S-doped graphene)	200 mAh g^{-1}	0.01 – 3.0 V	570 mAh g^{-1}	491 mAh g^{-1} (150th cycle)	this work

14 Zhang, C., Mahmood, N., Yin, H., Liu, F. & Hou, Y. Synthesis of Phosphorus-Doped

Graphene and its Multifunctional Applications for Oxygen Reduction Reaction and Lithium Ion Batteries. *Adv. Mater.* **25**, 4932-4937 (2013).

26 Li, X. *et al.* Superior cycle stability of nitrogen-doped graphene nanosheets as anodes for lithium ion batteries. *Electrochem. Comm.* **13**, 822-825 (2011).

30 Pan, D. *et al.* Li Storage Properties of Disordered Graphene Nanosheets. *Chem. Mater.* **21**, 3136-3142 (2009).

31 Yoo, E. *et al.* Large Reversible Li Storage of Graphene Nanosheet Families for Use in Rechargeable Lithium Ion Batteries. *Nano Lett.* **8**, 2277-2282 (2008).

32 Wu, Z.-S., Ren, W., Xu, L., Li, F. & Cheng, H.-M. Doped Graphene Sheets As Anode Materials with Superhigh Rate and Large Capacity for Lithium Ion Batteries. *ACS Nano* **5**, 5463-5471 (2011).

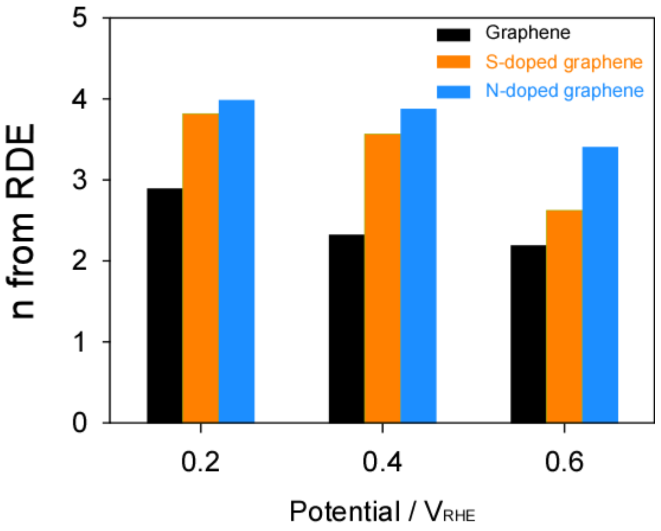

Supplementary Figure S7: Potential dependent electron transfer number of the pristine solvothermal graphene, N-doped graphene and S-doped graphene.
